# Sequential Delivery of Cryogel Released Growth Factors and Cytokines Accelerates Wound Healing and Improves Tissue Regeneration

**DOI:** 10.3389/fbioe.2020.00345

**Published:** 2020-04-17

**Authors:** Shiro Jimi, Alexandr Jaguparov, Ayan Nurkesh, Bolat Sultankulov, Arman Saparov

**Affiliations:** ^1^Central Laboratory for Pathology and Morphology, Faculty of Medicine, Fukuoka University, Fukuoka, Japan; ^2^Department of Medicine, School of Medicine, Nazarbayev University, Nur-Sultan, Kazakhstan

**Keywords:** biomaterials, cryogel, controlled release, growth factors, cytokines, wound healing, tissue regeneration

## Abstract

Growth factors and cytokines that are secreted by cells play a crucial role in the complex physiological reaction to tissue injury. The ability to spatially and temporally control their actions to maximize regenerative benefits and minimize side effects will help accelerate wound healing and improve tissue regeneration. In this study, the sequential targeted delivery of growth factor/cytokine combinations with regulatory functions on inflammation and tissue regeneration was examined using an internal splint wound healing model. Four examined growth factors and cytokines were effectively incorporated into a novel chitosan-based cryogel, which offered a controlled and sustained release of all factors while maintaining their biological activities. The cryogels incorporated with inflammation modulatory factors (IL-10 and TGF-β) and with wound healing factors (VEGF and FGF) were placed on the wound surface on day 0 and day 3, respectively, after wound initiation. Although wound area gradually decreased in all groups over time, the area in the cryogel group with growth factor/cytokine combinations was significantly reduced starting on day 7 and reached about 10% on day 10, as compared to 60–65% in the control groups. Sequential delivery of inflammation modulatory and wound healing factors enhanced granulation tissue formation, as well as functional neovascularization, leading to regenerative epithelialization. Collectively, the chitosan-based cryogel can serve as a controlled release system for sequential delivery of several growth factors and cytokines to accelerate tissue repair and regeneration.

## Introduction

Wound healing is a complex and well-orchestrated response to tissue injury and consists of four overlapping stages: hemostasis, inflammation, proliferation, and remodeling (Sun et al., 2014; Rodrigues et al., 2018). During tissue injury, the damaged cells release damage-associated molecular patterns and their intracellular contents that activate inflammasome and lead to the secretion of pro-inflammatory cytokines IL-1β and IL-18 (Saparov et al., 2017; Brazil et al., 2019). Platelets (PLTs) come in contact with the extracellular matrix and become activated, releasing clotting factors, promoting production of the matrix for migrating inflammatory cells, as well as various growth factors such as platelet-derived growth factor (PDGF), IL-1, TGF-α, and TGF-β that participate in wound healing (Saghazadeh et al., 2018; Brazil et al., 2019). PDGF, IL-1, IL-18, and other pro-inflammatory cytokines attract neutrophils to the injury site where they secrete proteases, reactive oxygen species, and phagocytose cell debris and pathogens, if present (Jorch and Kubes, 2017). Moreover, recruited neutrophils secrete TNF-α, IL-1, IL-6, and chemokines that stimulate migration of the cells of the immune system, including monocytes (Castano et al., 2018). Recruited monocytes differentiate into either pro-inflammatory macrophages, which produce TNF-α, IL-1β, IL-6, and IL-8, or reparative macrophages, which secrete IL-10 and TGF-β (Brazil et al., 2019). Macrophages also phagocytose the remaining debris and apoptotic neutrophils, as well as stimulate fibroblast infiltration. Migrated fibroblasts produce matrix metalloproteinases that facilitate the removal of disorganized extracellular matrix and create space for newly created components of the extracellular matrix (Castano et al., 2018). TGF-β is a proliferation and differentiation factor of fibroblasts, which differentiate into myofibroblasts that close the area of injury (Martin and Nunan, 2015; Brazil et al., 2019). In addition, VEGF, along with FGF, leads to the proliferation of endothelial cells and thus angiogenesis, an essential and integral part of the proliferative phase during which new blood vessels form (Sun et al., 2014). This allows oxygen and nutrients to be delivered to the site of injury, lack of which may hinder the process of wound healing (Castano et al., 2018). EGF, TGF−α, and FGF promote epithelial cell motility and proliferation, leading to re-epithelization (Arwert et al., 2012; Cordeiro and Jacinto, 2013).

In the past few decades, controllable drug delivery systems have been developed in order to effectively deliver various cytokines and growth factors to improve wound healing and support tissue regeneration. The most common types are polymeric micro and nanospheres, lipid nanoparticles, nanofibrous structures, coacervate, hydrogels, and scaffolds (Kakkar et al., 2017; Mansurov et al., 2017; Wang et al., 2017; Chen et al., 2018). Various polymer compositions of these delivery systems have demonstrated their potential in future therapeutic treatments, especially for tissue repair and regeneration (Saghazadeh et al., 2018; Chouhan et al., 2019). In this regard, the effects of various cytokines and growth factors on wound healing using various biomaterials have been studied. For instance, hEGF incorporated into a heparin-based hydrogel sheet demonstrated enhanced wound healing, granulation tissue and capillary formation, and epithelialization (Goh et al., 2016). TGF-β incorporated in layered scaffolds reduced the production of pro-inflammatory cytokines TNF-α, IL-12, and MCP-1 and decreased leukocyte migration post-implant (Liu et al., 2016). Moreover, it has also been shown that a combination of IL-4, IL-10, and TGF-β promotes an anti-inflammatory and pro-healing environment (Riabov et al., 2017). Other cytokines that have been well studied and show wound healing effects are FGF and VEGF. One study shows that wound healing is accelerated when the wound is exposed to a scaffold containing both FGF and VEGF (Losi et al., 2013). With the exception of hydrogels and polymeric scaffolds with high adsorbents capacity, one major disadvantage of such delivery systems for wound healing is the requirement for additional dressing, which could adsorb released exudates. Hydrogels are widely used; however, their non-porous structure could limit their use due to its semi-permeability to gases and water vapors in addition to poor mechanical stability and a poor bacterial barrier (Mayet et al., 2014).

Another recent development in drug delivery systems is cryogel, which is a modified form of hydrogel with the advantage of manipulating pore sizes, allowing for the passage of blood vessels and cells (Leach et al., 2019; Shah et al., 2019; Sultankulov et al., 2019b). It can be fabricated from both natural and synthetic polymers and has a wide range of tissue engineering applications, including bioreactors (Berillo et al., 2019), cell separation (Kumar and Srivastava, 2010), skin (Allan et al., 2016), cartilage (Han et al., 2016), bone (Kemençe and Bölgen, 2017; Sultankulov et al., 2019a), neural (Singh et al., 2018), and muscle tissue repair (Leach et al., 2019; Shah et al., 2019). Moreover, cryogels could be made using biopolymers with intrinsic antimicrobial activity, such as chitosan (Khan et al., 2019). Koshy et al. (2018) demonstrated that injectable cryogel is able to incorporate and release various molecules to mediate their biological activities. Based on published data of the anti-inflammatory and pro-healing properties of IL-10 and TGF-β as well as the angiogenic and regenerative properties of VEGF and FGF, these growth factors and cytokines (GF/C) were used in the current study. Thus, we hypothesized that sequential delivery of IL-10/TGF-β and FGF/VEGF incorporated into a controlled release system would modify the inflammation and proliferation stages of wound healing in order to accelerate wound healing and improve tissue regeneration in the internal splint wound model. To test this hypothesis, we utilized a novel chitosan-based cryogel that was composed of chitosan and heparin, and possessed high porosity and interconnective pore morphology. The chitosan-based cryogel was previously characterized and demonstrated the ability to incorporate and release bone morphogenic protein 2 for supporting the differentiation of mesenchymal stem cells into the osteogenic lineage (Sultankulov et al., 2019a). Chitosan is a positively charged polymer with the ability to form a polyelectrolyte complex with heparin, which makes incorporation of factors possible due to electrostatic interactions. The cryogels were successfully loaded with either inflammation modulatory factors (IL-10/TGF-β) or wound healing factors (FGF/VEGF) in order to regulate post-injury inflammation and tissue regeneration. The release profile of loaded factors and their biological activities *in vitro* and *in vivo*, as well as the ability to improve wound healing and tissue regeneration, were investigated.

## Materials and Methods

### Preparation of Cryogel

Cryogels were prepared as previously described (Sultankulov et al., 2019a). Briefly, 2% w/v Chitosan (Sigma, United States) was solubilized in 1% v/v acetic acid (Millipore, United States) and 5% w/v polyvinyl alcohol (PVA, Sigma, United States) was dissolved in deionized H_2_O on a heating plate using a magnetic stirrer for 1–2 h. For the preparation of cryogel, 5 ml of PVA, 5 ml of heparin (Sigma, United States), 10 ml of chitosan and 0.5% v/v glutaraldehyde (GA) (Sigma, United States) were sequentially added and mixed using a magnetic stirrer. After addition of GA, the final mixture was quickly aliquoted into 3 ml pre-cooled syringes and placed in a −12°C refrigerated circulator (Julabo F-34, Julabo, Germany) with subsequent incubation for 12–24 h. Then the cryogels were defrosted, washed with water, and incubated with 50 mM sodium borohydride (NaBH_4_) for 3 h on a benchtop shaker to neutralize GA (Migneault et al., 2004; Ivanov and Ljunggren, 2019). After any excess water was removed, cryogels were frozen at −20°C and lyophilized for 12–24 h (LTE Scientific, United Kingdom). Dried samples were stored at room temperature in a sealed tube until use.

### Incorporation and Release of GF/C

Cryogel samples (0.5–0.8 cm in diameter) were sliced into 3 mm thick disks, sterilized in 70% ethanol for 20 min, and placed under a fume hood UV light for 40 min. The following recombinant GF/C were used: IL-10 (Gibco, United States), TGF-β1 (Gibco, United States), VEGF-165 (Invitrogen, United States), and FGF2 (Invitrogen, United States). The samples were divided into the following seven groups: (1) cryogel incubated with PBS; (2) cryogel incubated with 1 μg/ml of IL-10 and TGF-β1; (3) cryogel incubated with 0.3 μg/ml of IL-10 and TGF-β1; (4) cryogel incubated with 0.1 μg/ml of IL-10 and TGF-β1; (5) cryogel incubated with 10 μg/ml of VEGF-165 and FGF2; (6) cryogel incubated with 3 μg/ml of VEGF-165 and FGF2; and (7) cryogel incubated with 1 μg/ml of VEGF-165 and FGF2. After incubation for 4 h at room temperature, the samples were centrifuged at 4000 *g* for 10 min to remove unincorporated factors. Then the cryogels were washed three times with PBS, dried, and left for incubation at 37°C in PBS supplemented with 10,000 U/ml of lysozyme (Sigma, United States). For release profile measurements, the supernatants were collected at 1, 3, 5, 7, and 10 day intervals and stored at −20°C.

### Detection of GF/C by ELISA

ELISA was performed according to the manufacturer’s protocols for VEGF, IL-10, FGF2, TGF-β1 (All Invitrogen, United States). Three different concentrations of each GF/C incorporated into cryogel were tested in duplicates and the absorbance was measured at 450 nm (Mithras LB 940, Berthold, Germany).

### Cell Lines

Mouse mast cells, MC/9 (ATCC, United States), mouse mammary gland cells, NMuMG (ATCC, United States), and mouse fibroblasts, NIH/3T3 (ATCC, United States) were maintained in DMEM with 4.5 g/l glucose, 2.0 mM L-glutamine, sodium pyruvate, and sodium bicarbonate (Sigma, United States), supplemented with 10% fetal bovine serum (FBS) and 1% penicillin/streptomycin (Sigma, United States). In addition, 4.0 mM L-glutamine, 10% Rat T-STIM with Con A (Corning, United States), and 0.05 mM 2-mercaptoethanol (Sigma, United States) were added to MC/9 cells and 10 μg/ml insulin (Sigma, United States) was added to NMuMG cells. Human umbilical vein endothelial cells (HUVECs) (Sigma, United States) were cultured in M199 (Sigma, United States) medium supplemented with 10% FBS (Gibco, United States).

### Biological Activity of FGF

To determine FGF biological activity, CytoSelect 24-well Wound Healing Assay (Cell Biolabs, United States) was used. The experiments were performed according to the manufacturer’s protocol. Briefly, inserts of 0.9 mm wound field were placed into previously collagen-coated [50 μg/ml in 0.1 M acetic acid (Millipore, United States)] 24-well plate. 10 × 10^5^ of NIH/3T3 mouse fibroblasts in 500 μl low-glucose DMEM (Sigma, United States) with 10% FBS (Sigma, United States) and 1% of penicillin/streptomycin (Sigma, United States) were incubated at 37°C overnight. The following three groups were used for the experiments: PBS, 50 ng/ml of FGF (Invitrogen, United States), and FGF released from the cryogel (day 3). Images were retrieved at 0 h and then every 2 h using EVOS^TM^ FL Auto 2 Imaging System (Invitrogen, United States). After 24 h, cells were fixed with 4% formaldehyde and stained with DAPI. Images were obtained and the surface area of the migrated cells was measured using Fiji software.

### Biological Activity of VEGF

Human umbilical vein endothelial cell proliferation was measured by Alamar Blue cell viability reagent (ThermoFisher Scientific, United States) according to the manufacturer’s protocol. Briefly, 1 × 10^4^ cells/well were seeded in a 96-well plate (TPP, Switzerland) and after the cells adhered, they were treated with 10 ng/ml VEGF (Sigma, United States), cryogel released VEGF after 3 days of incubation and PBS. Then, 10 μl/well Alamar Blue reagent was added to the cells and after incubation for 4 h, the absorbance in each well was measured at 570 nm. The cell proliferation level of the control or test groups was averaged from six independent wells.

### Biological Activity of IL-10

To determine the biological activity of IL-10, proliferation of MC/9 cells was measured. 5 × 10^3^ of MC/9 cells in culture media were treated with 50 ng/ml IL-4 (Sigma, United States), 25 ng/ml IL-10 (Gibco, United States), 50 ng/ml IL-4 plus 25 ng/ml IL-10, supernatant from cryogel (day 3), and supernatant from cryogel plus 50 ng/ml IL-4. Cells were incubated at 37°C for 2 days. After incubation, 10 μl of 3-(4,5-dimethylthiazol-2-yl)-2,5-diphenyl tetrazolium bromide (MTT solution) was added to each well. Upon 4–6 h incubation at 37°C, 100 μl of 10% SDS in 0.01 M HCl was added to each well and the absorbance was measured at 570 nm using a spectrophotometer (Varioskan Flash, Thermo Scientific, United States).

### Biological Activity of TGF-β

To determine the biological activity of TGF-β, NMuMG cells were used. Briefly, 5 × 10^3^ of NMuMG cells per well were treated with PBS, 2 ng/ml TGF-β (Gibco, United States), and cryogel released TGF-β (day 3). Cells were incubated at 37°C for 30–48 h and MTT assay was performed. 10 μl MTT solution was added to each well and incubated for 4–6 h. Finally, 100 μl of isopropanol in 0.04 M HCl was added to each well and the absorbance was measured at 570 nm using a spectrophotometer (Varioskan Flash, Thermo Scientific, United States).

### Animals

All animal experiments were approved by the Fukuoka University Animal Experiment Committee (No. 1706061). C57BL/6N female mice (Japan SLC, Shizuoka, Japan) at 6–10 weeks of age were used. All procedures were conducted under aseptic conditions. Mice were anesthetized with isoflurane (Wako Pure Chemical Industries, Ltd., Osaka, Japan) or pentobarbital (Somnopentyl, Kyoritsu Seiyaku, Tokyo, Japan). Hematological analyses, including red blood cell (RBC), white blood cell (WBC), and PLT counts (Celltac-α, Nihon Kohden, Tokyo, Japan) were performed with blood collected from the orbital sinus with a heparinized 75-μl capillary (Hirschmann Laborgeräte GmbH & Co., Eberstadt, Germany). At the end of study, the mice were sacrificed by lethal pentobarbital injection and arterial hemorrhage, and wound tissue was obtained.

### Internal Splint Wound Model

To create a humanized skin wound in the mouse, the previously published internal splint method was used (Jimi et al., 2017). In brief, mice were anesthetized with pentobarbital, and dorsal hair was removed with a commercial depilatory. A circular tattoo (1 cm in diameter) was made at the center of the lumbar area. The part of skin was completely excised with scissors, and a doughnut-shaped plastic splint (outer diameter: 22 mm, inner hole diameter: 16 mm) was inserted beneath the skin near the wound defect and attached to the fascia with six-stitch ligations. The splint was then fixed to the skin with surgical silk thread (six stitches at regular intervals). The wound surface was treated with 70% ethanol for 1 min to damage surficial cells on the wounded tissue, mimicking a deterred clinical wound with necrotic tissue. Finally, the splinted wound was covered with a polyurethane film dressing (Tegaderm, Sumitomo 3M, Tokyo, Japan). To prevent thread removal by the mice, they were dressed and fixed with a silicon-tight vest.

### Wound Treatments With Cryogel and GF/C

Cylinder-shaped freeze-dried cryogels were cut into 1 mm thick disks and immersed in a mixture of the following GF/C: IL-10 (1 μg/ml) and TGF-β1 (1 μg/ml) or VEGF (10 μg/ml) and FGF (10 μg/ml), and incubated at room temperature for 2 h on a shaker. After incubation, each cryogel was placed into a 24-well plate with 1 ml PBS, and then transferred into a cylinder of 2.5 ml syringe (Terumo Co., Tokyo, Japan) with a cap and 2 ml PBS was poured for subsequent incubation for 15 min. After incubation, PBS was drained and squeezed out. This procedure was repeated four times to wash out all free GF/C. The GF/C (IL-10/TGF-β1)-bound cryogel was minced with a surgical knife and placed on the wound surface just after wounding (day 0). The cryogel with VEGF/FGF was similarly placed on the wound surface on day 3 on top of the previously placed cryogel either with or without IL-10/TGF-β1.

### Wound Analysis

#### Macroscopic Analysis

Wounds were traced on a transparent plastic sheet on days 0, 3, 7, and 10. The wound area was measured using a computer-assisted morphometrical analyzer (VH Analyzer, VH-H1A5, Keyence Co., Osaka, Japan), and each wound area was calculated as a percentage of the wound area on day 0.

#### Microscopic Analysis

Dorsal skin tissue was dissected from the sacrificed mice. This tissue, together with the splint, was fixed in 10% buffered formaldehyde (pH 7.4) for several days. Two cross-cut tissue samples from each wound (about 5 mm thick) were excised. Paraffin blocks were prepared by using a tissue processor (Tissue-Tec VIP Premier, Sakura, Nagano, Japan), following which 4-μm-thick tissue sections were cut with a microtome (RM2235, Leica Biosystems, Nußloch, Germany).

### Histological Staining

Paraffin sections were stained with hematoxylin and eosin (HE) and Masson’s trichrome (MT). For immunohistochemical analysis, rabbit anti-α-smooth muscle actin antibody (αSMA: Abcam plc, Tokyo, Japan), rabbit anti-macrophage antibody (Mac-1: CD11b, Abcam plc), rat anti-neutrophil antibody (NINP-R14: Hycult Biotech, Uden, Netherlands), rat anti-CD31 antibody (Dianova GmbH, Hamburg, Germany), and rat anti-PI3 kinase p110 beta antibody (PI3K: Abcam plc) were used. Heat-antigen-activations in 0.01 M citrate buffer (pH 6.0) for CD31 antibody and Tris/EDTA buffer (pH9) for PI3K antibody were performed. For rabbit antibodies, Envison kit (Dako Japan Inc., Tokyo, Japan), and for rat antibodies, N-Histofine Simple Stain Mouse MAX PO (rat) detection system (Nichirei Bioscience Inc., Tokyo, Japan), were used. Colorization was performed with 3,3-diaminobenzidine. Hematoxylin was also used for a nucleus counter stain.

### Morphometric Analysis

#### Epithelial Length From the Wound Edge

Total epithelialization length (TEL) was defined as the length of epithelial growth from the dermal edge cut (above the splint hole) after wounding and measured according to the previous published method (Jimi et al., 2017). Two types of epithelial lengths were measured: the contracted epithelium length (CEL) and the regenerative epithelium length (REL). The TEL was the sum of CEL and REL. The CE was characterized by normal dermal structures with hair follicles and a dermal-muscular coat, whereas the RE grown on granulation tissue did not have these characteristics.

#### Granulation Thickness

The wound surface was gradually covered by proliferative granulation tissues; however, granulation thickness varied in different portions. Granulation tissue formed beneath the non-epithelialized zone, and beside the edge of the epidermal tissue, which must be highly responsible for epidermal regeneration. Zonal granulation tissue (about 445 μm in length in a picture captured with a 20 × objective lens) on the wound was selected; length and area of the granulation tissue were morphometrically measured. The thickness of the granulation tissue was calculated with the following formula: thickness (μm) = area (μm^2^)/length (μm).

#### Collagenosis

Four μm granulation sections stained with MT were used. As a reference color, blue color found in the collagen-rich fascia in one section was adopted for the collagen analysis in all of the granulation tissues. On the base of the reference color, the areas of similar blue staining with identical color range were measured. Collagenosis in the granulation was evaluated with the following formula: collagenosis (%) = collagen positive area/granulation area × 100.

#### Neovascularization

Magnified pictures (714 times) of granulation tissue stained with CD31 antibody were printed, and on which a transparent sheet with longitudinal multi-lines (7 μm distance by original size) was placed. The portions of CD31-positive sites that intersected with the grid lines were marked, and the total number of positive spots on each line was calculated, which represented the extent of neovascularization at a certain depth of the granulation. In some studies, the depth of granulation tissue was divided by 70 μm interval and designated as shallow (grid lines #1–10), middle (grid lines #11–20), and deep (grid lines #21–30).

### Laser Doppler Perfusion Image

On day 10, animals were anesthetized with isoflurane and the blood flow on the wound was analyzed with Doppler perfusion image device (Moor LDPI-2, Moor Instruments, Axminster, Devon, United Kingdom) with the beam probe (830 nm) positioned at 30 cm distance from the mice, and blood flow perfusion images were captured.

### Statistical Analysis

To compare two values, student’s *t*-test or Mann–Whitney *U*-test was performed. For multifactor analysis, one or two-way ANOVA was performed. *P*-values < 0.05 were considered to denote statistical significance. Data are expressed as mean values ± SD and *n* = 3, unless otherwise specified.

## Results

### *In vitro* Release of GF/C Incorporated Into Cryogel

**FIGURE 1:**
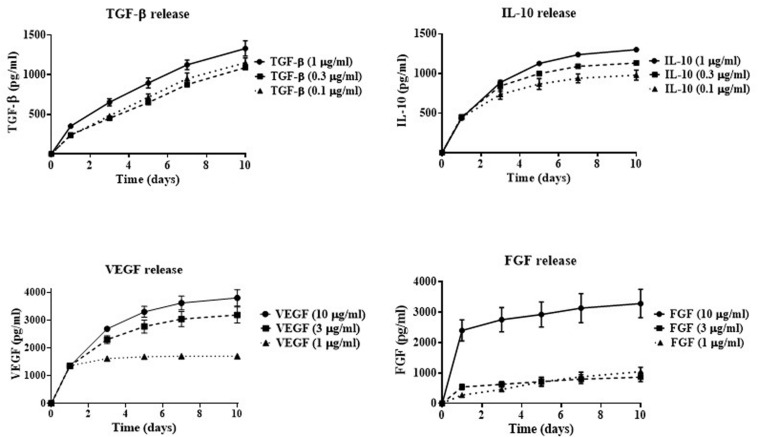
*In vitro* cumulative release of growth factors. Indicated concentrations of TGF-β, IL-10, VEGF, and FGF were incorporated into the cryogel. The concentrations of released growth factors in the solution were measured by ELISA at 1, 3, 5, 7, and 10 days after factor incorporation into the cryogel.

In order to determine the effectiveness of cryogel as a drug delivery system, GF/C release rate was investigated over a ten-day period. The following concentrations of four GF/C were used for this purpose: 1, 0.3, and 0.1 μg/ml of TGF-β and IL-10 or 10, 3, and 1 μg/ml of VEGF and FGF. The formation of polyelectrolyte complexes between oppositely charged groups of chitosan and heparin makes incorporation of factors possible due to electrostatic interactions (Mateescu et al., 2015; Minsky et al., 2017). After successful incorporation of the GF/C, the cryogels were washed several times to remove unincorporated GF/C and incubated at 37°C with 10,000 U/ml of lysozyme (Wang et al., 2013) to mimic the environment at the site of tissue injury where lysozyme can degrade chitosan (Freier et al., 2005; Foster et al., 2015). Then, the supernatants were collected at days 1, 3, 5, 7, and 10 and the concentrations of the released factors were measured using ELISA. According to our data (Figure 1), the cryogel demonstrates the release of GF/C in a controlled manner. The cumulative release of TGF-β increases gradually for all used concentrations with the highest release at 1 μg/ml. A similar release profile was demonstrated for IL-10 with the highest release rate at 1 μg/ml as well. In contrast, the two highest concentrations of incorporated VEGF (10 and 3 μg/ml) showed the highest release rate, while the lowest concentration plateaued after day 1. Similar results were obtained for the FGF release profile, when only the highest concentration (10 μg/ml) showed a gradual increase, while the two lowest concentrations plateaued after day 1 (Figure 1). Thus, our data demonstrate that cryogel possesses the ability to incorporate and gradually release all four GF/C, particularly at the highest concentration. Based on their release profiles, the highest concentrations of all GF/C were used in subsequent experiments.

### Biological Activity of GF/C

**FIGURE 2:**
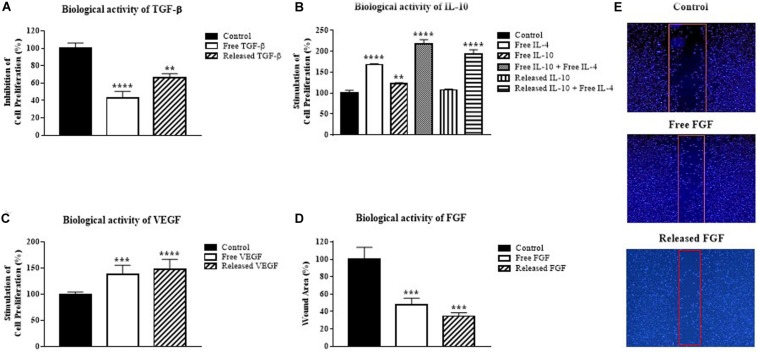
Biological activity of growth factors released from the cryogel. **(A)** Inhibition of mouse mammary gland cell line (NMuMG) proliferation by the cryogel released TGF-β, **(B)** Stimulation of mouse mast cell line (MC/9) proliferation by the cryogel released IL-10, **(C)** Stimulation of human umbilical vein endothelial cells (HUVEC) proliferation by the cryogel released VEGF, **(D)** Decrease in the wound area by the cryogel released FGF. **(E)** Representative images of wound areas quantified in **(D)**. Data were analyzed using one-way ANOVA with Bonferroni’s multiple comparisons test. **P < 0.01, ***P < 0.001, ****P < 0.0001 compared with the control group.

To examine the biological activity of incorporated GF/C *in vitro*, the supernatants collected at day 3 post incorporation were used to perform cell proliferation assays with an appropriate test model for each GF/C. For TGF-β, epithelial mouse mammary gland (NMuMG) cell line was used for the ability of TGF-β to suppress proliferation of these cells (Xie et al., 2003). The addition of free exogenous TGF-β significantly suppressed proliferation (57%) of NMuMG cells. Similarly, cryogel released TGF-β also inhibited proliferation (34%) of this cell line (Figure 2A). These data indicate that cryogel released TFG-β is biologically active.

MC/9 mast cells were used to test the biological activity of IL-10 in combination with IL-4 based on its effectiveness as a model, as previously described (Thompson-Snipes et al., 1991). The addition of either IL-4 or IL-10 alone increased MC/9 mast cell proliferation by 68 and 23%, respectively (Figure 2B). As expected, the combination of both IL-10 and IL-4 increased MC/9 cell proliferation more than twofold (118%). A similar increase in cell proliferation was demonstrated by cryogel released IL-10 in combination with free IL-4 (93%). These results show that IL-10 incorporated into cryogel possesses biological activity.

The biological activity of the released VEGF was tested on HUVECs (Marinval et al., 2018). Treatment of HUVEC cells with VEGF demonstrated that released VEGF stimulates cell proliferation as effectively as free VEGF (Figure 2C). Thus, VEGF released from the cryogel has biological activity.

To test the biological activity of FGF, NIH/3T3 fibroblast cell line was used for its ability to respond to FGF (Igarashi et al., 1998). The *in vitro* wound healing assay with 0.9 mm inserts was used to test the biological activity of FGF. After overnight incubation, released FGF treated fibroblasts proliferated and moved toward the uncultured gap side as quickly as the free FGF group. Significant wound area healing up to 53% for free FGF and 65% for released FGF was detected in comparison to the control group (Figures 2D,E). In all experiments, the amounts of released GF/C on day 3 were smaller than the added exogenous concentrations. However, the smaller amounts of the released GF/C were still able to mediate their biological effects. Taken together, our data indicate that all GF/C released from the cryogel possess biological activity *in vitro*.

### Effects on Wound Healing

**FIGURE 3:**
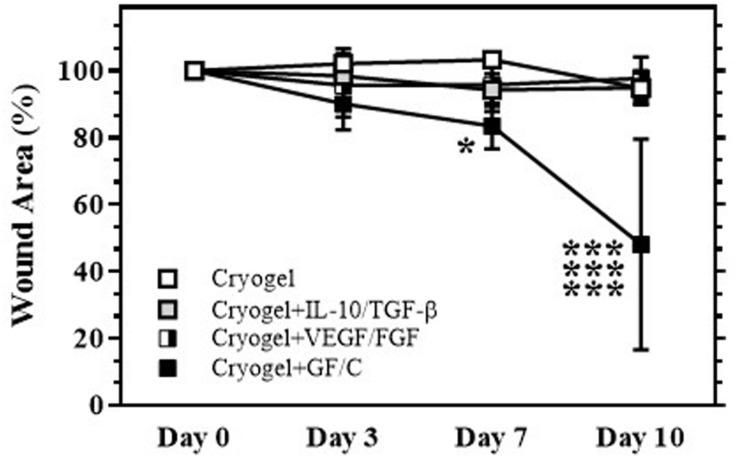
Wound healing after treatment with cryogel. After total skin excision in mice, an internal splint model was performed, and the mice were divided into four groups by wound treatment: treated with cryogel alone, cryogel plus IL-10/TGF-β, cryogel plus VEGF/FGF, and cryogel plus all growth factors/cytokines (GF/C). Changes in wound area (% vs. day 0) over time are shown. Wound areas in the group with cryogel plus GF/C significantly decreased on days 7 and 10 as compared to the other groups. Values: mean ± SE. ***P < 0.01 compared to other groups, *P < 0.05 compared to cryogel alone group.

The internal splint model established by our group (Jimi et al., 2017) that was located on the back was used in this study. To determine whether individual or sequential treatments with cryogel plus GF/C affect wound healing, the mice were divided into the following four groups: treated with cryogel alone on days 0 and 3 after wound initiation (Group with cryogel); treated with cryogel containing TGF-β and IL-10 on day 0 and with cryogel alone on day 3 (Group with cryogel plus TGF-β/IL-10); treated with cryogel alone on day 0 and with the cryogel containing VEGF and FGF on day 3 (Group with cryogel plus VEGF/FGF); treated with cryogel containing TGF-β and IL-10 on day 0 and with cryogel containing VEGF and FGF on day 3 (Group with cryogel plus GF/C). Our data indicate that there is no statistically significant difference between groups treated with cryogel alone and treated individually with either TGF-β/IL-10 or VEGF/FGF (Figure 3). However, the sequential treatment with combinations of GF/C significantly reduced wound area compared to all groups. In addition, the treatment with just solutions of TGF-β/IL-10 on day 0 and VEGF/FGF on day 3 did not improve wound healing (data not shown). It is likely that proteolytic enzymes, which are present in the wound area, degraded GF/C that were delivered in the solutions (Choi et al., 2018; Li et al., 2019). Taken together, our data demonstrate that sequential treatment with the cryogels containing combinations of GF/C improves the wound healing. Based on the results of these experiments, only the group with sequential treatment of GF/C containing cryogels was used in subsequent experiments.

**FIGURE 4:**
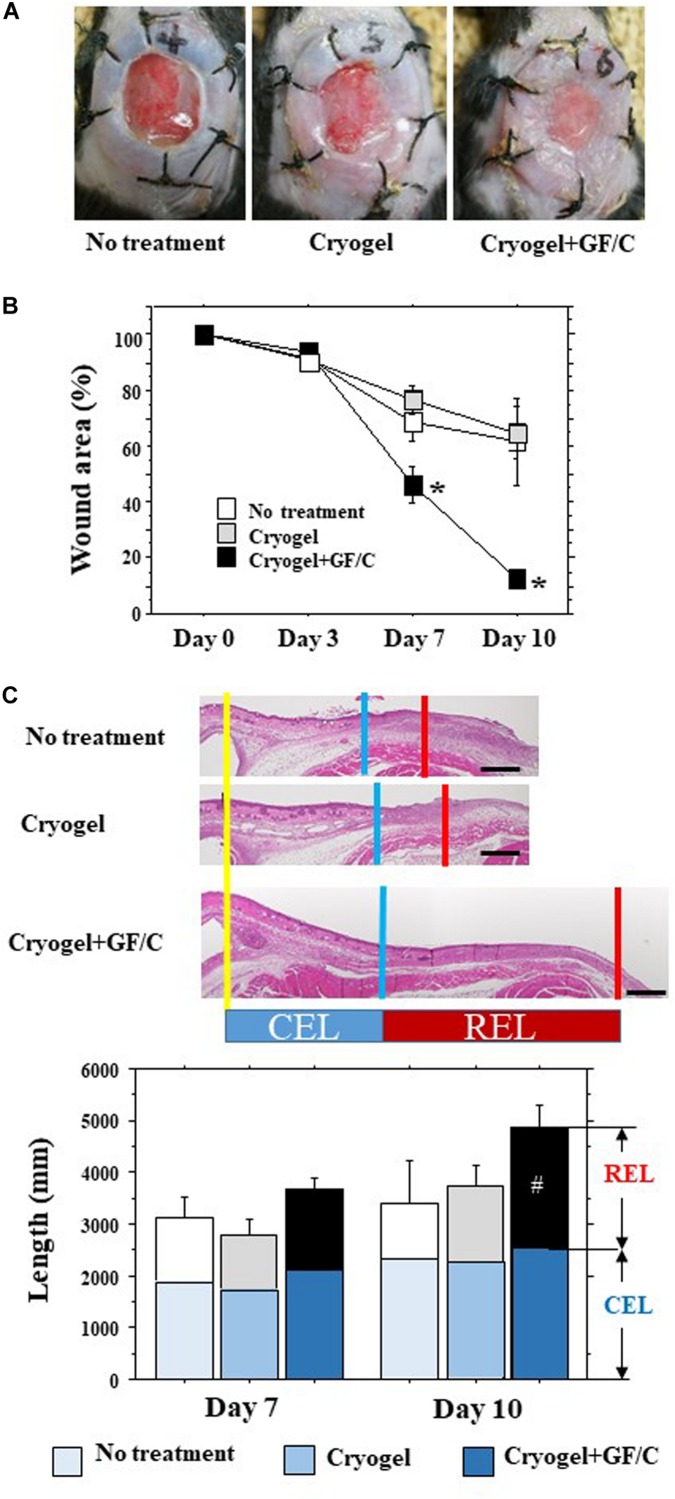
Wound healing and epithelial regeneration. After total skin excision in mice, an internal splint model was performed, and the mice were divided into three groups by wound treatment: no treatment, treated with cryogel alone and cryogel plus growth factors/cytokines (GF/C). **(A)** Representative pictures of the wounds in the groups with no treatment, treated with cryogel alone and cryogel plus GF/C on day 10. The wounds were covered with whitish tissue that extended from the skin margin. **(B)** Changes in wound area (% vs. day 0) over time. Wound areas in the group with cryogel plus GF/C significantly decreased on days 7 and 10 as compared to the other groups. **(C)** Epithelialization of wound surface. Epithelialization was evaluated as CEL (contracted epithelium length) and regenerative epithelium length (REL). Total epithelial length (CEL + REL) increased with time in all groups, in which no changes were found in CEL. REL in the group with cryogel plus GF/C on day 10 was significantly extended as compared to the group with no treatment. *n* = 5. Bar = 100 μm. Values: mean ± SE. *P < 0.05 compared to the no treatment group and group with cryogel alone, #*P* < 0.05 compared to the no treatment group.

For the second set of experiments on the effects on wound healing, the mice were divided into the following three groups: a control group with no treatment, a group to which cryogel alone was applied to the site of the wound on days 0 and 3 (Group with cryogel), and a group that was placed with the cryogel containing TGF-β and IL-10 on day 0 and with the cryogel containing VEGF and FGF on day 3 after initiation of the wound (Group with cryogel plus GF/C). Representative pictures of the wounds in the groups with no treatment, treated with cryogel alone and cryogel plus GF/C on day 10 are shown in Figure 4A. The wound area after total skin excision reduced over time (Figure 4B). On day 10, the wound area reduced to about 60–65% in the groups with no treatment and cryogel alone, whereas the group with cryogel plus GF/C demonstrated reduction to 13%. As a result, the wound area in the group with cryogel plus GF/C was significantly smaller compared to groups with no treatment and cryogel alone. When transversal sections of the wounds were examined (Figure 4C), splint holes and epidermal healings were identical. CEL for the contractive length of the epidermis, REL for the regenerative length of the epidermis, and TEL for total epithelial length were measured (Figure 4C). On days 7 and 10, no differences were found in CEL and TEL among the groups; however, REL in the group with cryogel plus GF/C on day 10 was significantly elongated as compared to the group with no treatment.

### Granulation Tissue Development and Collagenosis

**FIGURE 5:**
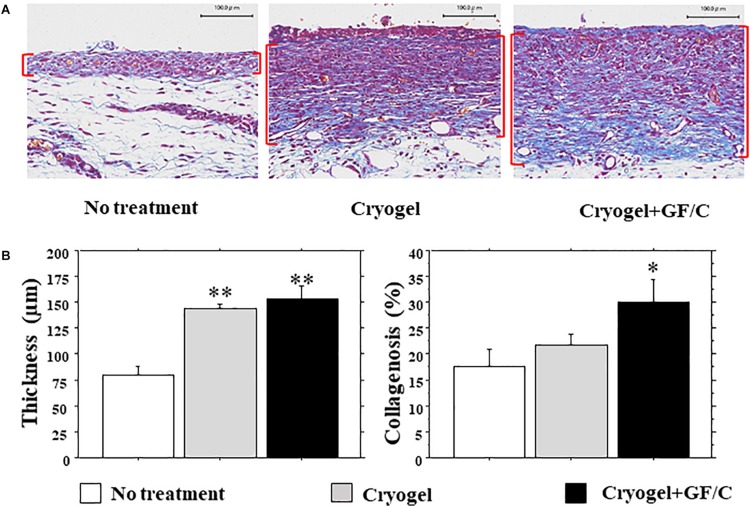
Development of granulation tissue and collagenosis. **(A)** Representative images of granulation tissues (parenthesized in red) stained with MT in each group. **(B)** Thickness and collagenosis in the granulation tissues. The values were evaluated with a morphometrical method. Granulations in the groups with cryogel alone and cryogel plus GF/C were significantly thicker than in the group with no treatment. Whereas collagenosis (blue in MT staining) in the group with cryogel plus GF/C progressed significantly as compared to the group with no treatment. *n* = 5. Bar = 100 μm. Values: mean ± SE. *P < 0.05, **P < 0.01 compared to the no treatment group.

The wound surface was covered by proliferative granulation tissues; however, granulation thickness was different at different portions. We therefore focused on the granulation beside the front of the growing epidermis (Figure 5A), which may be highly responsible for epidermal regeneration. The granulation thickness in groups with cryogel alone and cryogel plus GF/C on day 10 was significantly greater than in the group with no treatment (Figure 5B, left panel). Collagenosis detected by blue color in MT stain was also measured in the granulation tissue. Collagenosis was significantly advanced in the group with cryogel plus GF/C as compared to the group with no treatment. However, no difference was detected between groups with no treatment and cryogel alone (Figure 5B, right panel).

### Neovascularization and Blood Flow

**FIGURE 6:**
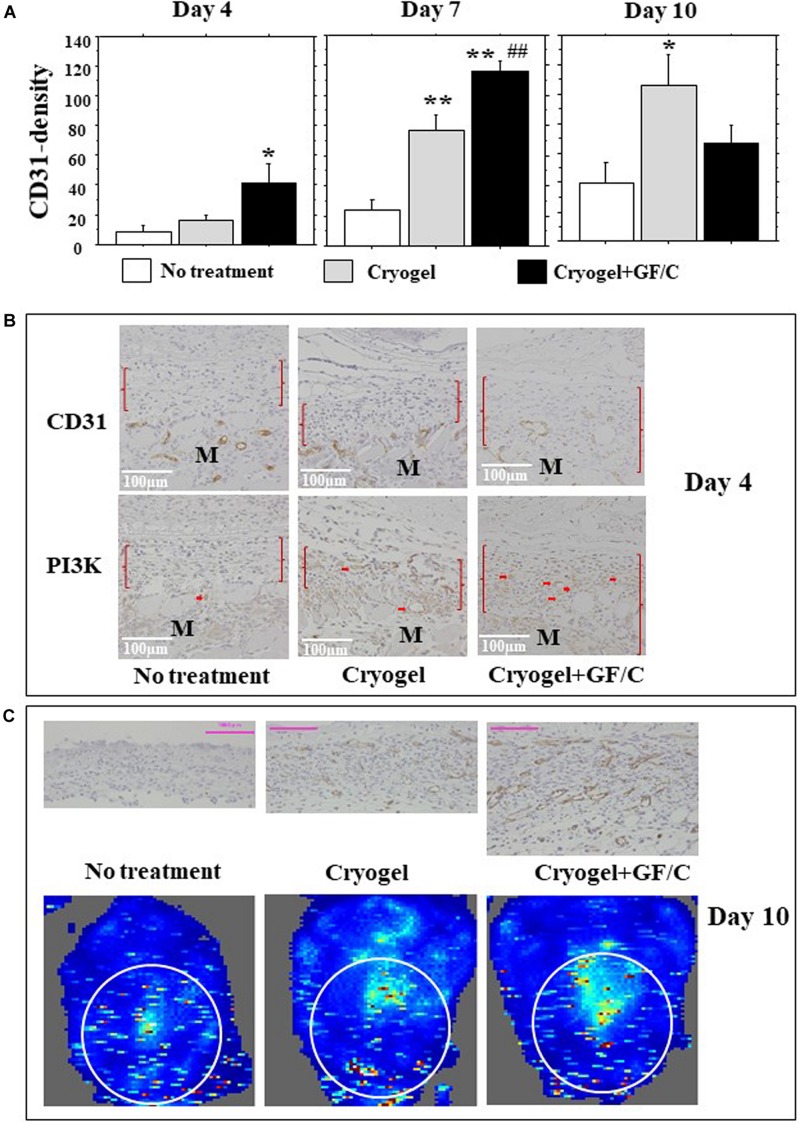
Neovascularization and blood flow images. **(A)** The number of CD31-positive endothelial cells was measured in the granulation tissue and was considered as neovascularization. The density of CD31 positive cells increased on day 4 only in the group with cryogel plus GF/C, and was greater on day 7 in the group with cryogel plus GF/C than the groups with no treatment and cryogel alone, and was also greater in the group with cryogel alone compared to the group with no treatment. On day 10, the density increased in groups with no treatment and cryogel alone, but decreased in the group with cryogel plus GF/C. **(B)** Representative images of CD31 and PI3K staining are shown using serial sections. PI3K positive cells were mainly found in migrated vessels (arrows) only in the group with cryogel plus GF/C. M—muscle tissue. **(C)** The blood flow Doppler images indicated chromaticity on wound surface, and its corresponding tissues in cross-section were stained with CD31 antibody on day 10. Values: mean ± SE. *n* = 5. Bar = 100 μm. *P < 0.05, **P < 0.01 compared to the group with no treatment. ##*P* < 0.01 compared to the group with cryogel alone.

Neovascularization occurs during tissue granulation. In this study, capillaries grew primarily in longitudinal directions. For this reason, CD31-positive cell densities were measured using a horizontal grid. On day 4, few positive cells were found in the group with no treatment, and the number of CD31 positive cells in the group with cryogel plus GF/C increased significantly. On day 7, the number of cells in the groups with cryogel and cryogel plus GF/C increased significantly as compared to the group with no treatment (Figure 6A). Moreover, the number of CD31 positive cells in the group with cryogel plus GF/C was significantly greater than the group with cryogel alone. The data obtained on day 10 revealed that the CD31-positive density in the groups with no treatment and cryogel alone progressively increased, but decreased in the group with cryogel plus GF/C. Intracellular pathway of VEGF was examined next. On day 4, PI3K was detected in the migrated vessels in the granulation only in the group with cryogel plus GF/C (Figure 6B). As shown in Figure 6C (upper panel), neovessels in the group with cryogel plus GF/C are wide and dense, showing that the vessels are well matured. Doppler images of blood flow on the wounds were analyzed. Our data demonstrate that the image distribution was highest in the group with cryogel plus GF/C (42.1 +2.8), followed by the group with cryogel alone (18.6 +4.3) and the group with no treatment (22.3 +1.1) (Figure 6C, low panel).

### Neovascularization in Depth of Granulation

**FIGURE 7:**
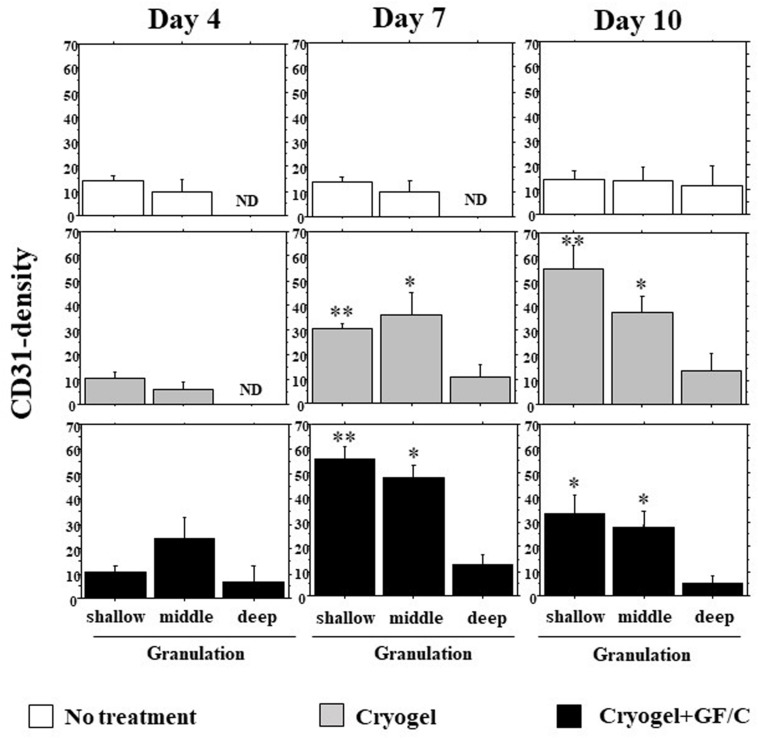
Development of neovascularization in the granulation tissue. The number of CD31 positive cells in the granulation tissue depending on depth was analyzed on days 4, 7, and 10. On day 7, neovascularization was not found in the group with no treatment; however, it progressed in the groups with cryogel alone and cryogel plus GF/C. This reaction was highest in the group with cryogel plus GF/C, and showed the shape of downhill stairs. Values: mean ± SE. *n* = 5. ND—not determined, *P < 0.05, **P < 0.01 compared to the no treatment group.

Neovascularization depending on the depth of the granulations on days 4, 7, and 10 was analyzed (original results are shown in Supplementary Figure S1). The following criteria were used to define the depth as shallow (between surface and 70 μm depth), middle (70–140 μm), and deep (140–210 μm). On day 4, neovascularization started from the bottom of granulation in all three groups. But, in the group with cryogel plus GF/C, neovessels tended to be dense in the middle depth. In the group with no treatment, fewer vessel density was found on day 7. On day 10, the density increased slightly, but the levels were still flat in different depths (Figure 7). In the group with cryogel on day 7, the density moderately increased, especially in the shallow and middle depths. On day 10, the appearance of the density pattern was in the shape of downhill stairs. In the group with cryogel plus GF/C, this shape already appeared as early as day 7. On day 10, the pattern was unchanged, but all of the density decreased.

## Discussion

Cells of the immune system and their secreted factors play a crucial role in tissue damage and regeneration following a wound injury (Chung et al., 2017; Julier et al., 2017). Production of reactive oxygen species by the migrated neutrophils further damages the wounded tissue and leads to tissue damage and cell death. Migrated monocytes clean the cell debris by phagocytosis, produce various factors, and participate in tissue regeneration. Other cells and their secreted factors are also involved in this complex and well-orchestrated process of tissue damage and healing (Bosurgi et al., 2017; Eming et al., 2017; Dahlgren and Molofsky, 2019). There is a fine balance between the detrimental and beneficial effects of the cells of the immune system during inflammation. Excessive inflammation leads to extended tissue damage that is difficult to heal, while very limited inflammation leads to improper tissue repair and regeneration (Dahlgren and Molofsky, 2019). One approach for improving wound healing is to limit inflammation and aid in accelerating tissue repair and regeneration. In the present study, a novel chitosan-based cryogel was used for two key reasons. First, chitosan-based biomaterials possess water-holding and antimicrobial properties and are biodegradable, which make it suitable for improving wound healing (Zhao et al., 2017; Chen et al., 2018). Second, various GF/C can be incorporated into cryogel due to positively and negatively charged residues, which will allow local and sustained release of bound factors to avoid the adverse effects of delivery of large amounts of GF/C. TGF-β and IL-10 were chosen for their ability to regulate immune response and suppress inflammation. In contrast, VEGF and FGF were selected for their ability to stimulate neovascularization and tissue regeneration (Yu et al., 2017; Niu et al., 2018; Xue et al., 2018). All incorporated GF/C exhibit a sustained release profile with gradual increase in the amounts of released factors. The released factors were able to mediate their desirable biological activities *in vitro*: TGF-β suppressed the proliferation of epithelial mouse mammary gland (NMuMG) cell line, IL-10 in combination with IL-4 stimulated the proliferation of MC/9 mast cells, VEGF stimulated the proliferation of HUVECs, and FGF improved *in vitro* wound healing.

For the experimental splint wound healing model, the cryogel was placed on the muscular fasciae following total skin excision. There was some evidence of a foreign-body reaction with phagocytic cell infiltration, including macrophages and neutrophils, but the mass gradually dissolved/phagocytosed and assimilated with the granulation tissue. Moreover, αSMA-positive myofibroblast infiltration and CD31-positive neovascularization took place in the granulation tissue (Supplementary Figure S2). In addition, no cryogel fragments remained in the granulation tissue on day 7 (4 days after the last cryogel placement), which was crucial for epithelization. Dissolution of cryogel fragments may help to avoid continuous inflammation and prevent chronic inflammation and extensive scar formation.

Granulation is formed to provisionally fill the tissue at the site of defect and if healing is completed, then the tissue regresses (Rousselle et al., 2018). A newly formed granulation tissue provides a scaffold for migrated cells to mediate their effects, which are essential for wound healing (You et al., 2017). In our study, we focused on the zone of the granulation tissue beside the edge of epithelialization, which may support subsequent epidermal extension. It has been shown that chitosan-based hydrogels are effective in wound healing (Hamedi et al., 2018); however, the exact mechanisms that are responsible for pathological changes are not defined yet. As compared to the untreated group of mice, granulation was greatly developed in both groups placed with the cryogel, independent of the presence of GF/C, during the 10 days of study. However, collagenosis advanced significantly only in the group with loaded GF/C as a result of granulation maturation. Although cryogel itself performed as a favorable substrate for granulation development, it failed to achieve tissue epithelialization. Thus, our data indicate that GF/C released from cryogel contribute to wound healing. Ballestas et al. (2019) recently reported that nanofiber scaffolds improve wound healing by increasing recruitment of LyC6^*lo*^ monocytes and generating reparative M2 macrophages, as well as elevating expression of IL-10 and reducing levels of pro-inflammatory cytokines such as IL-1, IL-4, and IL-6. A similar importance of M2 macrophages in improving wound healing was shown by using scaffolds that contain JK1, a controllable hydrogen sulfide donor (Wu et al., 2019). Similar to IL-10, TGF-β released from scaffolds also suppressed inflammation and reduced expression of MCP-1 and pro-inflammatory cytokines such as TNF-α and IL-12 (Liu et al., 2016).

Neovascularization during the development of granulation is an important step in wound healing (Rousselle et al., 2018). Newly formed vascular beds can provide blood supply, including oxygen and nutrients, to the regenerating tissues. In this study, granulation is developed on the exposed muscular fascia, and fibroblast proliferation and endothelial extension may take place during granulation. The direction of newly formed vessels therefore revealed an upward distribution in the cross-sectional granulation tissue. Furthermore, bone marrow-derived endothelial progenitor cells (EPCs) also participate in neovascularization (Shi et al., 2017; Lu and Li, 2018). Many CD31-positive single cells were found in the granulation tissue on day 7, which may include endothelial cells originated from EPC. Nevertheless, neovascularization found in this study occurred via angiogenesis, as well as vasculogenesis (Qadura et al., 2018), resulting in the creation of favorable conditions for cell migration, which is created by the GF/C released from the cryogel. It was shown that both FGF and VEGF released from biomaterials improve wound healing. FGF increases the density of the newly formed and matured vessel, as well as improves re-epithelization and ECM regeneration (Liu et al., 2017), whereas VEGF can induce the re-programming of monocytes to become angiogenic and arteriogenic (Avraham-Davidi et al., 2013).

**FIGURE 8:**
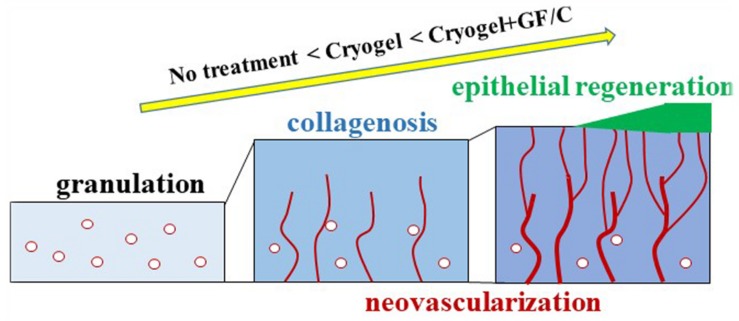
Wound healing progression. At the site of skin wounding, granulation tissue developed. This reaction aims to initiate neovascularization to supply blood derived factors as well as oxygen and nutrients, to regenerate epithelium. During granulation development accompanied with deposition of collagen fibers, granulation tissue acts as a scaffold for cell migration and proliferation. Newly formed vessels derived from the fascia and/or EPCs migrate in an upward direction in the granulation. Connected vessels finally reach the site at which epithelial regeneration take place. This process advances in the order of no treatment < cryogel alone < cryogel plus GF/C as demonstrated in the present study.

CD31-positive neovascularization in the granulation tissue was evaluated by means of morphometrical technique. In the negative control group and the group treated with cryogel only, CD31 density increased over time, which reflects the development of neovascularization. Whereas the density in the group treated with cryogel plus GF/C was highest on day 7, and subsequently decreased despite the increase in epithelialization on day 10. This discrepancy is likely since blood supply to the regenerative epithelium, as compared to other groups, was efficient as shown by the laser Doppler. During establishment of blood supply, unnecessary vessels may be discarded. According to the distribution analysis of vessel density in the depths of granulation, it is also obvious that a stepwise change in graph was found in the group treated with cryogel plus GF/C on day 7, and the highest density was shown in the shallow part. This tendency still remained on day 10. A similar distribution pattern also appeared later in the group treated with cryogel only on day 10. These results indicate that when compared to the group without any treatment, functional neovascularization was established early by the cryogel plus GF/C group, and the group treated with cryogel only followed thereafter. One of the mechanisms by which VEGF stimulates neovascularization is the activation of intracellular PI3K pathway as early as day 4. These findings are summarized in Figure 8. Thus, wound healing progresses as a common physiological tissue reaction; however, cryogel treatment accelerates this reaction, whereas GF/C associated with the cryogel are even more effective. We believe that both inflammation modulatory factors (IL-10 and TGF-β) and wound healing factors (VEGF and FGF) are crucial for modulating inflammation to accelerate wound healing and improve tissue regeneration. However, inflammation modulatory factors are more useful for a clinical translation because without modulating inflammation, it is not possible to accelerate wound healing and improve tissue regeneration.

## Conclusion

The present study first shows the pathophysiological effects of cryogels on wound healing. Cryogels acted as a substrate or scaffold not only for GF/C, but also for tissue cells, which resulted in the acceleration of wound healing. Moreover, cryogel serves as an effective drug delivery system with sustainable release of incorporated GF/C and dissolves several days after placement *in vivo*. These findings suggest that cryogels have high biocompatibility and are safe, and enhance granulation tissue formation. In particular, functional neovascularization leading to regenerative epithelialization could be improved by cryogels. Therefore, cryogels may have a potential for practical and functional wound healing applications as an accelerating material/carrier. Taken together, cryogels can be used for wound healing treatments in clinical practice in the future.

## Data Availability Statement

All datasets generated for this study are included in the article/Supplementary Material.

## Ethics Statement

All animal experiments were approved by the Fukuoka University Animal Experiment Committee (No. 1706061).

## Author Contributions

AS contributed to conception and design of the study. SJ, AJ, AN, and BS performed the experiments and analyzed the data. SJ, AJ, AN, and AS wrote sections of the manuscript. AS wrote the first draft of the manuscript. All authors contributed to manuscript revision and read and approved the submitted version.

## Conflict of Interest

Patent application for the preparation of CHI-PVA-Hep-GA has been submitted in the Republic of Kazakhstan. The authors declare that the research was conducted in the absence of any commercial or financial relationships that could be construed as a potential conflict of interest.
