# Supplementary material for: Sequential Delivery of Cryogel Released Growth Factors and Cytokines Accelerates Wound Healing and Improves Tissue Regeneration
Source: Front Bioeng Biotechnol. 2020 Apr 17;8:345. doi: 10.3389/fbioe.2020.00345 (PMC7212449; doi:10.3389/fbioe.2020.00345)
Supplement: Supplementary file 1 [file Table_1.DOCX]

**Supplementary Figure 1.** CD31-positive neovascularization density in varying depths of the granulation tissue in each mouse in groups with no treatment, cryogel alone and cryogel plus growth factors on days 4, 7 and 10.

**Supplementary Figure 2. Cryogel and granulation tissue reaction.** The serial sections of a specimen from the cryogel plus GF group on Day 10. Granulation developed after placing a cryogel mass onto the wound surface. Cryogel fibers are strongly stained with hematoxylin and eosin (HE stain), and thus highlighted in yellow. In the granulation tissue, cryogel fibers are fragmented and remained in the granulation, where collagenous matrix (blue area in MT staining) was accompanied with neovascularization (CD31-positive endothelium) and aSMA-positive vascular walls, and aSMA-positive myofibroblast also accumulated in the matrix. Whereas polymorphonuclear neutrophils (PMNs) and macrophages (Mac1) were infiltrated to some extent around the cryogels as a foreign-body reaction.
